# Near-Field and Far-Field Directional Conversion of Spoof Surface Plasmon Polaritons

**DOI:** 10.1038/srep33496

**Published:** 2016-09-15

**Authors:** Heng-He Tang, Yunhua Tan, Pu-Kun Liu

**Affiliations:** 1 School of Electronics Engineering and Computer Science, Peking University, Beijing, 100871, China

## Abstract

A compact metallic meta-structure is proposed to realize directional conversion between spoof surface plasmon polaritons (SSPPs) and propagating waves at millimeter wave and THz frequencies. The structure is constructed by embedding two slits or multi-slits array into a subwavelength metallic reflection grating. When the back-side of the structure is illuminated by an oblique beam with a fixed incident angle, the propagating wave will be unidirectionally converted into SSPPs with a considerable efficiency. Both the simulations and experiments demonstrate that the excitation ratio of the SSPPs between the two possible propagating directions (left and right) reaches up to about 340. Furthermore, assisted by the structure, near-field SSPPs can be also converted into far-field narrow beams with particular directions. Through frequency sweeping, wide-angle beam scanning is verified by theory and experiments. The work paves a new way for SSPPs launching and also provides fresh ideas for super-resolution imaging in the longer wavelength range.

In the recent years, structures with surface topology engineering^1^,^2^,^3^,^4^,^5^ have been widely employed to manipulate near-field waves in the microwave to terahertz (THz) frequency range^6^. For example, deep localized surface waves can be supported on those metallic structures with subwavelength periodic engraved holes or grooves^7^,^8^. Because the surface waves have lots in common with the famous surface plasmon polaritons (SPPs)^9^,^10^,^11^,^12^ in the optical frequency range, they are frequently called as spoof surface plasmon polaritons (SSPPs)^13^,^14^,^15^,^16^,^17^,^18^. SSPPs can be also used for non-diffraction limited waveguiding^19^ and super-resolution imaging^20^ in the longer wavelength range owing to their subwavelength cross sections of localized fields. One of our recent reports also suggests SSPPs to be used for designing new compact and efficient THz sources^21^. Even though SSPPs can be simply excited by putting a probe with a subwavelength tip closed to the structures^22^,^23^ or etching a single narrow slit inside the structures^24^,^25^, these traditional ways both suffer from the problem of inherent low efficiency. In ref. 26, a new SSPPs excitation scheme based on a transparent gradient metasurface is reported to achieve a 73% measured efficiency. However, investigations on how to directionally launch SSPPs with considerable efficiencies are not currently underway. Potentially, directional launching of SSPPs with tunability are highly demanded in various applications such as SSPPs-based THz sources^21^ and ultra-compact THz integrated circuits^27^. By placing two optimized gratings on the opposite sides of a narrow slit, SSPPs at different frequencies can be separately guided in two desired directions^28^. However, the propagation direction of SSPPs at a fixed frequency cannot be tunable. In the optical frequency range, unidirectional launching of SPPs can be realized by Bragg reflection originating from a periodic array of grooves that carved into a metal film^29^, or by near-field interference of surface waves emerging from polarization-sensitive apertures^30^ and one or two slits^31^,^32^,^33^,^34^,^35^. Despite of the low launching efficiency, the two slits coupling method can be also suggested in the unidirectional launching of SSPPs in the longer wavelength range^36^.

In this paper, the unidirectional launching of SSPPs supported on a subwavelength metallic reflection grating is theoretically and experimentally demonstrated in the millimeter wave to THz frequency range. The launching efficiency via two slits coupling is greatly improved by embedding multi-slits array into the reflection grating. The tunable unidirectional launching of SSPPs emerges from the near-field constructive and destructive interference occurred only when the incident angle of the back-side illuminated beam and the period of the slits arrays are elaborately matched. We also find that SSPPs at different frequencies can be directionally converted into far-field narrow beams assisted by the structure, and beam scanning through frequency sweeping is also demonstrated by theory and experiments.

## Results

### Analytical theory of SSPPs

**Figure 1:**
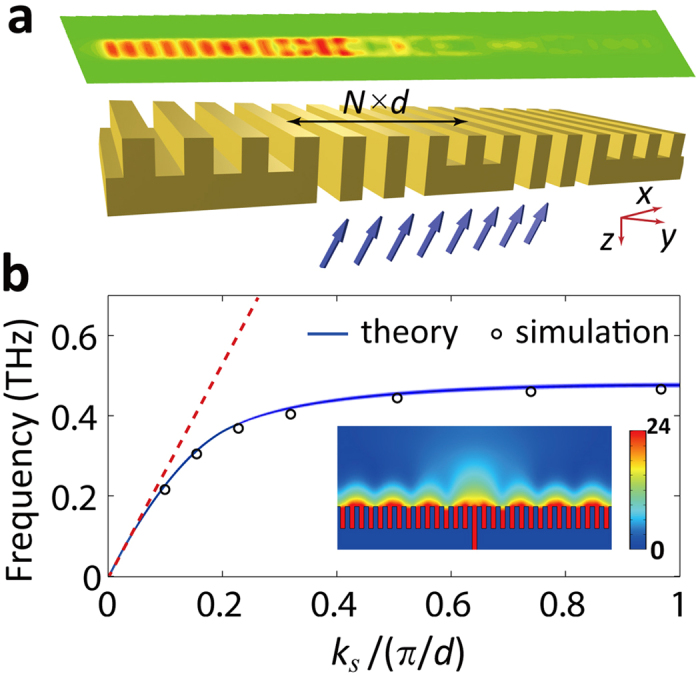
Schematic and dispersion of the structure. **(a)** Schematic view of the sturcture and unidirectional launching of SSPPs. The color map is the amplititude distribution of the electic field. **(b)** Dispersion of SSPPs supported on a reflection gratiing. The blue solid line is the theorectical resluts caculated from equation (1). The circles are the simulated results. The red dashed line is the light line. The figure presented in the inset is the magnetic field mappling of the SSPPs wave, which is coupling from a single narrow slit.

The schematic of our designed structure is illustrated in Fig. 1(a). By periodically replacing some adjacent grooves in a reflection grating with some slits, back-side incident waves can be coupled into the slits arrays, and then transformed into the SSPPs. For normal incidence, the excitation of SSPPs will be bidirectional due to the symmetry of the system, which means the localized surface waves will propagate toward both the left and right sides of the structure. Only when the constructive and destructive interferences of the surface waves emerging from different slits occur, the power flow will be guided in one way (left or right), which indicates the unidirectional launching of the SSPPs, as seen from Fig. 1(a).

The width and period of the grooves and slits are *a* and *d*, respectively. The heights of the grooves and slits are *h*_1_ and *h*_2_, respectively. Here, we assume there are *N* metallic walls in a basic unit of the periodic substitution area of the structure, in which the numbers of the metallic walls in the slit-arrays and groove-arrays are *N*_1_ and *N*_2_, respectively, *N* = *N*_1_ + *N*_2_. Hence, the period of the basic units can be expressed as *N* × *d*. The total number of the basic units is *M*. When to find out the optimal constructive and destructive interference conditions, investigations on the dispersion of SSPPs supported on the reflection grating must be firstly implemented. The modal expansion method (MEM)^33^ is used to theoretically deduce the dispersion equation. For the theoretical analysis, we consider an infinite depth of the reflection grating in the *y* direction. When considering the continuity of the tangential components of the electric and magnetic fields at the boundary of grating-air interfaces, we get a transcendental equation describing the parallel momentum *k*_*s*_ of the SSPPs as a function of the frequency,


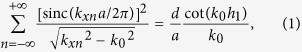


in which, *k*_0_ = *ω*/*c* is the wavenumber in air, *ω* is the angular frequency, *k*_*xn*_ = *k*_*s*_ + 2*n*π/*d*, *n* represents the diffraction order. Based on equation (1), we plot the dispersion curve in Fig. 1(b). The corresponding parameters of the grating are *d* = 60 *μm*, *a* = 30 *μm* and *h*_1_ = 140 *μm*. Note that, there is a big momentum mismatch between the SSPPs and free space waves especially for those highly localized SSPPs with extraordinary *k*_*s*_. Except for the forbidden bands, every frequency point corresponds to a particular eigen surface mode. The above analytical theory is also demonstrated by the full wave electromagnetic simulation. Here, the commercial software Comsol Multiphysics is employed. As shown in Fig. 1(b), there is a good agreement between the theoretical and simulating results. In the inset of Fig. 1(b), the simulated magnetic field distribution of SSPPs excited at 0.4 THz is also presented. For this case, we use a single slit back-side illumination method to excite the SSPPs, as we have predicted, the localized surface waves propagate toward both the two sides of the slit.

### Unidirectional launching of SSPPs

**Figure 2:**
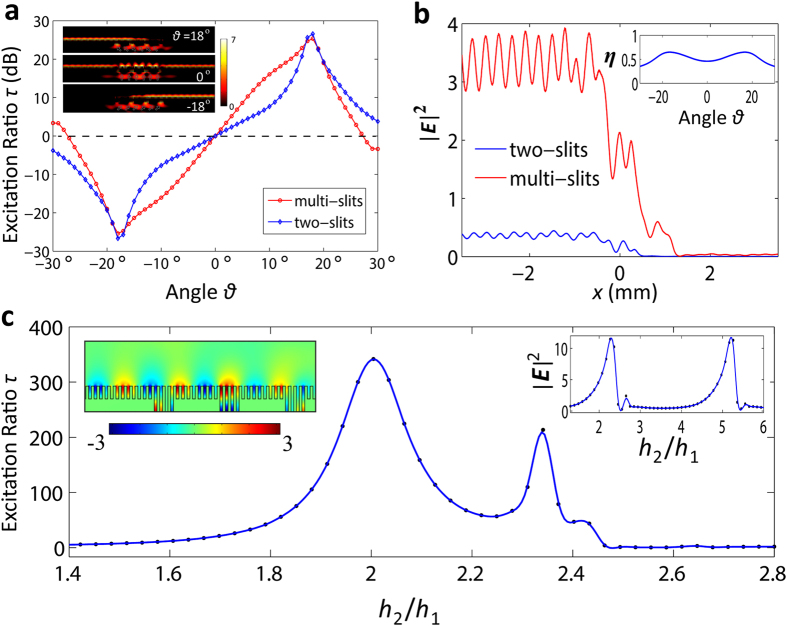
Simulated results of SSPPs launching. **(a)** The excitation ratio *τ* in dB as a function of the back-side incident angle. The red solid line marked with circles corresponds to the multi-slits array configuration. The blue solid line marked with rhombuses corresponds to the two-slits configuration. The simulated electric field mappings of the multi-slits configuration at *θ* = 0° and ±18°are presented in the inset. **(b)** Comparison of the SSPPs intensities coupling from the two slits (blue solid line) and multi-slits array (red solid line). The coupling efficiency *η* is presented in the inset. **(c)**The excitation ratio *τ* as a function of the height of the slits *h*_2_. The upper left inset, which illustrates the smooth surface mode transition, is the *x*-component of electric field distribution in the structure. The upper right inset is the square of electric field (|*E*|^2^) as a function of a broad-range *h*_2_. The field is detected 0.2 *mm* away from the upper interface.

Obviously, using a single coupling source can never have SSPPs interferences. If another slit is embedded in the grating, the separate launching of SSPPs from the two slits will establish near-field interference. The key point to fulfill the constructive or destructive interference condition is to modulate the phase differences between the two surface waves. For this purpose, an oblique incident beam must be in demand. The initial phase difference between the two surface waves, which is solely induced by the oblique illumination, is *k*_0_ *l* sin(*θ*), in which *θ* and *l* are the angle of the oblique incidence and the distance between the two slits, respectively. If we want the SSPPs to be unidirectionally guided toward left, then the constructive interference condition on the left side and the destructive interference condition on the right side can be respectively expressed by


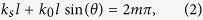



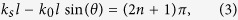


in which, *m* and *n* are arbitrary integers. Once the working frequency is known, *k*_*s*_ can be calculated by equation (1). Jointly solve equations (2) and (3), we can determine the values of *l* and *θ*. We should note that, the value of *l* can only be discrete due to the periodicity of the grating, which means *l* = *N* × *d*. So for most of working frequencies, the perfect constructive or destructive interference condition cannot meet. But because the period of the grooves in the grating is much smaller than the wavelength, equations (2) and (3) can be approximately satisfied. In this way, a simulated excitation ratio up to 340 can be got at *l* = 12*d*, *θ* = 18° when the working frequency is 0.365 THz, as shown in Fig. 2(a). The excitation ratio is calculated by |*E*_*l*_|^2^/|*E*_*r*_|^2^, where *E*_*l*_ and *E*_*r*_ are the amplitudes of electric fields detected at the left and right terminals of the structure, respectively. The two detection points are symmetric and 0.2 *mm* away from the upper surface of the structure. Note that, the excitation ratio will be constant when detecting the field at some arbitrary distances. The parameters of the basic unit in the two slits configuration are *N*_1_ = 0, *N*_2_ = 12, *M* = 2, the other parameters are *d* = 60 *μm*, *a* = 30 *μm*, *h*_1_ = 140 *μm* and *h*_2_ = 280 *μm*. It should be pointed out that, the launching efficiency of SSPPs via two slits coupling is not sufficient because of the very limited coupling area. Most of the incident energies are reflected away. This problem cannot be simply solved by widening the width of the slits. Actually, the SSPPs generation shows a sinusoidal dependence on slit width, which can be understood as a consequence of the diffractive nature of the slits^37^. The dominant forces of SSPPs excitation are evanescent-wave components coupling from the slits. By widening the width of the slits, the coupling energies in the slits are inclined to convert to far-field transmission waves rather than evanescent waves.

Our further studies show that the launching efficiency can be improved by using the multi-slits array configuration shown in Fig. 1(a). For this case, we also use equations (2) and (3) to determine the parameters of *θ* and *N*. The values of *N*_1_ and *M* are optimized by simulation. Our simulated results indicate that the height of the slits *h*_2_ in this case (i.e. *N*_1_ > 0, *M* > 2) has a great influence on the excitation ratio. As we know, SSPPs can be also supported on a transmission grating^38^. If we regard the multi-slits as a transmission grating with finite periods, then the influence of *h*_2_ can be simply comprehended as a problem of surface mode matching between the transmission and reflection gratings. We can also use the MEM to deduce the dispersion of the SSPPs supported on transmission gratings, which is expressed by


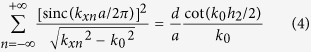


Comparing equation (1) with equation (4), we find that the two dispersion equations coincide exactly when *h*_2_ = 2*h*_1_, which indicates a smooth surface mode transition between the transmission and reflection gratings. Considering the symmetry of the transmission grating, the middle cut plane in the *z* direction is actually an electrical boundary. So the transmission grating can be equivalent to a back-to-back bonding of two reflection grating with zero substrate thickness, which also indicates the same conclusion of *h*_2_ = 2*h*_1_.

To illustrate the dependence of SSPPs launching on the incident angle *θ*, we use simulation to monitor the excitation ratio at the incident angles ranged from −30° to 30°. The working frequency of the simulation is 0.365 THz, the same as below. The parameters of the structure are *d* = 60 *μm*, *a* = 30 *μm*, *h*_2_ = 2*h*_1_ = 280 *μm*, *N* = 12, *N*_1_ = 3, *N*_2_ = 9, *M* = 4. The simulated results are shown in Fig. 2(a). With the increase of *θ*, the SSPPs can be tuned from bidirectional launching at *θ* = 0° (i.e. normal incidence) to unidirectional launching at *θ* = ±18°. As the theory predicted, both the two slits and multi-slits array configurations have a same unidirectional angle. The simulated electric field mappings at *θ* = 0° and ±18° are presented in inset of Fig. 2(a). The largest excitation ratio at *θ* = ±18° is |*τ|* = 25.28 dB.

In Fig. 2(b), we compare the electric field distributions of the SSPPs supported on the two slits and multi-slits array configurations. As we can note, the coupling power (corresponding to the coupling efficiency *η*) of the SSPPs supported on the multi-slits array configurations is enhanced by about 9 times. Here, the coupling efficiency is defined as the ratio of the SSPP energy to the total illumination energy on the back side, and is calculated by *η* = 1 − *r*_0_ − *t*_0_, in which *r*_0_ and *t*_0_ are the reflection and scattering coefficients, respectively. As illustrated in the inset of Fig. 2(b), we notice that, for the illumination angle ranged from −30° to 30°, the simulated average coupling efficiency is about 50%. At the unidirectional angles (i.e. ±18°), the coupling efficiency reaches to 65%.

At the optimal incident angle of unidirectional launching, i.e. *θ* = 18°, we plot the excitation ratio *τ* as a function of *h*_2_ in Fig. 2(c), from which we can see that *τ* reaches the maxima when *h*_2_/*h*_1_ = 2, as the above theory predicted. In the upper left inset of Fig. 2(c), the *x*-component of electric field at *h*_2_ = 2*h*_1_ = 280 *um* is presented. From the figure we can note that the profile and the parallel momentum of the SSPPs mode are maintained throughout the propagation along the surface of the structure. Due to the smooth surface mode transition, the unidirectionally propagating SSPPs will be hardly reflected back when they pass through the multi-slits, which eventually contributes the sharp resonance of *τ* at *h*_2_/*h*_1_ = 2.

As we see from Fig. 2(c), another resonance of the *τ* occurs at *h*_2_/*h*_1_ = 2.34. After deep investigations we find that it comes from the Fano resonance^39^,^40^ in each of the slits. When the width of the slits is much smaller than the wavelength, only TEM mode can be sustained in the slits. The field can be expressed by exp(−*ik*_0_*y*) + *r* ∙ exp(*ik*_0_*y*), which is a superposition of the forward and backward TEM mode, *r* is the reflection coefficient at the upper interface of the slits. The position of the Fano resonance is determined by *k*_0_*h*_2_ + Δ*ϕ*_*r*_ = *mπ*, where Δ*ϕ*_*r*_ is the phase shift caused by the reflection *r*^38^. In our configuration, as related to Fig. 1(a), the position of the Fano resonance is *h*_2F_ = *m*λ/2 − 2.68*a*, *m* are arbitrary integers, λ is the wavelength in air. In the upper right inset of Fig. 2(c), when *h*_2_/*h*_1_ is ranged from 1.3 to 6, we note two transmission peaks of the electric fields, which are consistent with the theoretically predicted Fano resonance positions. The electric fields are detected at the left terminal of the structure. Even though the coupling energies of the SSPPs are vastly enhanced at the resonance points, the moderate reflection of the SSPPs owing to the surface mode mismatch between the reflection grating and the multi-slits will degenerate the excitation ratio. That is why the optimal unidirectional launching of SSPPs does not happen at the Fano resonance points. However, if the launching efficiency is the point of focus, the Fano resonance becomes very helpful.

### Experimental verification of SSPPs launching

**Figure 3:**
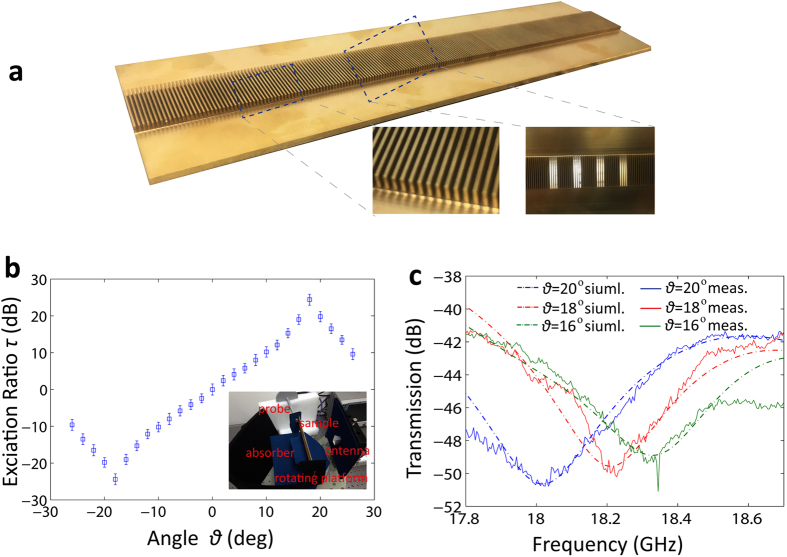
Experimental results of SSPPs launching. **(a)** The image of the fabricated sample, with two enlarged views being shown. **(b)** The measrued resluts of the excitation ratio in dB, with the error bars representing the testing uncertainty. The test scenario is shown in the inset. **(c)** Simulated (dash dotted lines) and measured (solid lines) transmissions at the destructive interference terminals.

Limited by the processing and testing capabilities we use a scaled model of the structure for the experimental demonstrations. This is all right because the mechanism of the SSPPs unidirectional launching is general applicable in the frequency range from microwave to THz. And whatever the working frequency is, the analytical theory indicates a same illumination angle dependence of the unidirectional launching for every scaled model. Here, the working frequency of the experiments is chosen to be 18.25 GHz. We only consider the multi-slits array configuration. The corresponding parameters of the structure are *d* = 1.2 *mm*, *a* = 0.6 *mm* and *h*_2_ = 2*h*_1_ = 5.6 *mm*, the other parameters keep no change. The image of the sample is shown in Fig. 3(a), accompanying with two enlarged views. The averaged measured results and estimated errors of the excitation ratio as a function of *θ* are shown in Fig. 3(b). As can be seen, the comparison between the theory and experiments is satisfactory. The test scenario is shown in the inset of Fig. 3(b).

Actually, when the parameters of the structure are determined, the working frequency of SSPPs unidirectional launching is not necessary to be fixed to a particular value. According to equations (2) and (3), if *l* is known, *k*_*s*_ (i.e. the working frequency) and *θ* can be solved. It means that an arbitrary illumination angle will correspond to a particular frequency point which satisfies the optimal SSPPs unidirectional launching. As shown in Fig. 3(c), we monitor the power transmission at the destructive interference terminals in the frequency range of 17.8 GHz to 18.7 GHz. When *θ* varied from 16° to 20°, the working frequency (corresponding to the valley points) has a redshift, as demonstrated by both the simulated and measured results.

### Far-field beam scanning

Through the multi-slits array coupling, the conversion from near-field surface waves to far-field propagating waves can be also achieved. For this case, the multi-slits array can be actually regarded as a radiation sources array which is fed by the SSPPs. The phase difference between the adjacent multi-slits is constant and can be continuously adjustable by changing the frequency. Hence, the emergence angle of the output propagating wave can be continuously tuned by frequency sweeping. When *d* and *h*_1_ are much smaller than the working wavelength, equation (1) can be simplified as *k*_*s*_^2^ = *k*_0_^2^[1 + tan^2^(*k*_0_*h*_1_)*a*/*d*]. So the emergence phase difference between the adjacent multi-slits, which can be expressed by *k*_*s*_*Nd*, gradually accumulates with the increase of frequency. In this way, the emergence angles are varied at different frequencies, which can be exactly known from


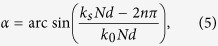


**Figure 4:**
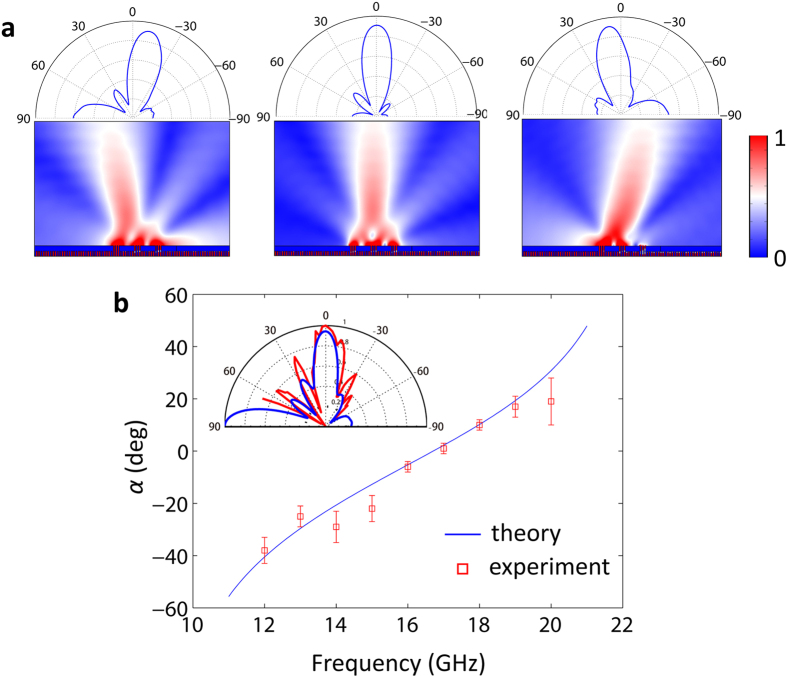
Far-field beam scanning. **(a)** Field mapping and far-field directional diagrams at three different frequencies *f* = 0.36 THz (left), 0.38 THz (middle) and 0.40 THz (right). The parameters of the structure are *N*_1_ = 2, *N*_2_ = 7, *M* = 3, *d* = 60 *μm*, *a* = 30 *μm* and *h*_2_ = 2*h*_1_ = 280 *μm*. **(b)** The theoretical (blue solid line) and experimental (red squares) emergence angles of the fabricated sample as a function of the frequency varied from 11GHz to 21 GHz. The far-field directional diagram at *f* = 16.5 GHz (corresponding to *α* = 0°) is presented in the inset, with the blue and red solid lines being the simulated and measured results, respectively. The parameters of the structure are same as Fig. 3(b,c). The error bars correspond to the testing uncertainty.

To check the validity of equation (5), we have carried out numerical calculations. The SSPPs are excited by a probe with its tip being 0.2 *mm* away from the grating surface. The SSPPs propagate along the grating surface from left to right. The parameters of the structure are *N*_1_ = 2, *N*_2_ = 7 and *M* = 3. The other parameters are same as in Fig. 3. We present the field mapping and far-field directional diagrams at three different frequencies *f* = 0.36 THz, 0.38 THz, 0.40 THz in Fig. 4(a). As we see, the corresponding orientations of the far-field beam at the three frequencies points are −10°, 0° and 10°, respectively. The theoretical predication of the emergence angles is also demonstrated by experiments. Here, the fabricated sample of the scaled model, shown in Fig. 3(a), is also employed. As we can see from Fig. 4(b), when the frequency is varied from 11 GHz to 21 GHz, the theoretical and measured emergence angles have a good agreement. Through frequency sweeping, wide-angle beam scanning from −60° to 60° is realized assisted by the structure. The simulated (blue solid line) and measured (red solid line) far-field directional diagram at 16.5 GHz (corresponding to the normal radiation) is presented in the inset of Fig. 4(b). The two results are also well matched.

## Discussion

Comparing those reported SPPs unidirectional launcher in the optical frequency range, the excitation ratio of SSPPs unidirectional launching in our proposal is competitive. The possible reasons come from two aspects. First, owing to the periodical perforations on the metal slab, the propagating directions of SSPPs are restricted to only left or right. For SPPs, the surface waves can propagate along all directions paralleling to the surface of metal slabs. Hence, deep constructive and destructive interferences can occur in SSPPs launching. Second, in the longer wavelength range, metals behave more like perfect conductors. Therefore, the losses of SSPPs are much smaller than the losses of SPPs. Except for the competitive excitation ratio, the significant 9 times improvement of the coupling efficiency of SSPPs by our proposed multi-slits array configuration makes the design becomes more attractive. The coupling efficiency can be further enhanced by adding the number of the multi-slits. But our simulated results show a degeneration of the excitation ratio with the increase of *M*. This is understandable given the more complex interferences of the surface waves. In practice, we should make a tradeoff between the coupling efficiency and the excitation ratio.

It should be pointed out that one of key technical problems to beat the diffraction limit in far-field is to convert near-field evanescent waves to far-field propagating waves^41^,^42^,^43^. As demonstrated above, our designed structure can actually perform in this way. So we hope the structure can pave new ways to realize far-field super-resolution imaging in the longer wavelength range. Through frequency sweeping^44^, the high spatial harmonics of targets (here it is 1.17*k*_0_~3.67*k*_0_) will excite the corresponding SSPPs modes. After the near-field to far-field conversion assisted by the structure, those harmonics information can be received at different angles in the far-field. Then the super-resolved images can be reconstructed. It should be pointed out that the near-field to far-field conversion of SSPPs, assisted by a periodically modulated 1D subwavelength corrugated metal structure, is also reported in ref. 45.

In conclusion, the directional conversion between SSPPs and propagating waves is theoretical and experimental demonstrated by our designed compact structure. Although, in terms of the coupling efficiency, our proposal shows no superiority over those proposals in previous literatures^16^,^26^, our work has successfully solved the problem of SSPPs unidirectional launching. The largest excitation ratio of 340 is obtained. The SSPPs launching can be tuned to be unidirectional or bidirectional. We also find that SSPPs at different frequencies can be directionally converted into far-field narrow beams assisted by the structure, and a wide-angle beam scanning from −60° to 60° is theoretical and experimental verified. The work paves a new way for SSPPs launching and also provides fresh ideas for super-resolution imaging in the longer wavelength range.

## Methods

Numerical simulations are performed by the commercial software, Comsol Multiphysics, which is based on the Finite Element Method (FEM). The material of the structure in the simulations is chosen to be Copper with the electric conductivity being 5.998e7 S/m. The scattering boundary condition is used. The oblique incident beam is generated by setting an electric field source. The *x*-component and *y*-component electric field can be respectively expressed by *E*_*x*_ = *g*(*x*)exp(−*ik*_*x*_*x* *−* *ik*_*y*_*y*)(−*k*_*x*_) and *E*_*y*_ = *g*(*x*)exp(−*ik*_*x*_*x* *−* *ik*_*y*_*y*)(*k*_*y*_), in which *g*(*x*) is a normalized Gaussian function with the standard deviation being 1.5e-3. The grooves and slits in the experimental structure are fabricated by electrosparking. We use Keysight Vector Network Analyzer (PNA-X, up to 50 GHz) to measure the transmission coefficients S21 of the fabricated sample. The test scenario is illustrated in the inset of Fig. 3(e). A rectangular horn antenna is used to produce the far-field illumination beam. The sample is back-side illuminated by the beam. The powers at the left and right terminals are detected by an open-ended rectangular waveguide probe (ORWP). The working frequency of the antenna and probe is ranged from 14.5 GHz to 22 GHz. The sample is mounted onto a motorized rotating stage. In this way, the angle of the illumination can be continuously and precisely changed. When testing the emergence angles of scanning beam and the far-field directional diagrams, we only need to exchange the positions of the horn antenna and ORWP.

## Additional Information

**How to cite this article**: Tang, H.-H. *et al.* Near-Field and Far-Field Directional Conversion of Spoof Surface Plasmon Polaritons. *Sci. Rep.* **6**, 33496; doi: 10.1038/srep33496 (2016).

## References

[b1] Sun S. *et al.* Gradient-index meta-surfaces as a bridge linking propagating waves and surface waves. Nat. Mat. 11, 426–431 (2012). 10.1038/nmat3292 22466746

[b2] Ma X. *et al.* A planar chiral meta-surface for optical vortex generation and focusing. Sci. Rep. 5, 10365 (2015). 25988213 10.1038/srep10365 PMC4437373

[b3] Unlu M. *et al.* Switchable scattering meta-surfaces for broadband terahertz modulation. Sci. Rep. 4, 5708 (2014). 25028123 10.1038/srep05708 PMC4099982

[b4] Li X. *et al.* Flat metasurfaces to focus electromagnetic waves in reflection geometry. Opt. Lett. 37, 4940–4942 (2012). 23202097 10.1364/OL.37.004940

[b5] Kuznetsov S. A., Astafev M. A., Beruete M. & Navarro-Cia M.. Planar holographic metasurfaces for terahertz focusing. Sci. Rep. 5, 7738 (2015). 25583565 10.1038/srep07738 PMC4291574

[b6] Tonouchi M. Cutting-edge terahertz technology. Nat. Photonics 1, 97–105 (2007).

[b7] Pendry J. B., Martin-Moreno L. & Garcia-Vidal. F. J. Mimicking surface plasmons with structured surfaces. Science 305, 847–848 (2004). 15247438 10.1126/science.1098999

[b8] Garcia-Vidal F. J., Martin-Moreno L. & Pendry. J. B. Surfaces with holes in them: new plasmonic metamaterials. J. Opt. A: Pure Appl. Op. 7, S97 (2005).

[b9] Barnes W. L., Dereux A. & Ebbesen T. W.. Surface plasmon subwavelength optics. Nature 424, 824–830 (2003). 12917696 10.1038/nature01937

[b10] Zayats A.V., Smolyaninov I. I. & Maradudin. A. A. Nano-optics of surface plasmon polaritons. Phys. Rep. 408, 131–314 (2005).

[b11] Liu Y. & Zhang. X. Metasurfaces for manipulating surface plasmons. Appl. Phys. Lett. 103, 141101 (2013).

[b12] Ditlbacher H., Krenn J. R., Schider G., Leitner A. & Aussenegg F. R.. Two-dimensional optics with surface plasmon polaritons. Appl. Phys. Lett. 81, 1762–1764 (2002).

[b13] Williams C. R. *et al.* Highly confined guiding of terahertz surface plasmon polaritons on structured metal surfaces. Nat. Photonics 2, 175–179 (2008).

[b14] Wang K. & Mittleman D. M.. Dispersion of surface plasmon polaritons on metal wires in the terahertz frequency range. Phys. Rev. Lett. 96, 157401 (2006). 16712193 10.1103/PhysRevLett.96.157401

[b15] Maier S. A., Andrews S. R., Martin-Moreno L. & Garcia-Vidal F. J.. Terahertz surface plasmon-polariton propagation and focusing on periodically corrugated metal wires. Phys. Rev. Lett. 97, 176805 (2006). 17155495 10.1103/PhysRevLett.97.176805

[b16] Ma H. F., Shen X., Cheng Q., Jiang W. X. & Cui T. J.. Broadband and high-efficiency conversion from guided waves to spoof surface plasmon polaritons. Laser & Photonics Rev. 8, 146–151 (2014).

[b17] Yu N. *et al.* Designer spoof surface plasmon structures collimate terahertz laser beams. Nat. Mat. 9, 730–735 (2010). 10.1038/nmat2822 20693995

[b18] Lockyear M. J., Hibbins A. P. & Sambles J. R.. Microwave surface-plasmon-like modes on thin metamaterials. Phys. Rev. Lett. 102, 073901 (2009). 19257669 10.1103/PhysRevLett.102.073901

[b19] Kumar G. *et al.* Planar plasmonic terahertz waveguides based on periodically corrugated metal films. New J. Phys. 13, 033024 (2011).

[b20] Tang H.-H. & Liu P.-K.. Terahertz far-field superresolution imaging through spoof surface plasmons illumination. Opt. Lett. 40, 5822–5825 (2015). 26670521 10.1364/OL.40.005822

[b21] Kong L.-B. *et al.* Enhancing spoof surface-plasmons with gradient metasurfaces. Sci. Rep. 5, 8772 (2015). 25740679 10.1038/srep08772 PMC4350089

[b22] Charbonneau R., Lisicka-Shrzek E. & Berini P.. Broadside coupling to long-range surface plasmons using an angle-cleaved optical fiber. Appl. Phys. Lett. 92, 101102 (2008).

[b23] Hanson G. W. *et al.* Excitation of terahertz surface plasmons on graphene surfaces by an elementary dipole and quantum emitter: Strong electrodynamic effect of dielectric support. Phys. Rev. B 86, 235440 (2012).

[b24] Lalanne P., Hugonin J. P. & Rodier J. C.. Theory of surface plasmon generation at nanoslit apertures. Phys. Rev. Lett. 95, 263902 (2005). 16486354 10.1103/PhysRevLett.95.263902

[b25] Laluet J. Y. *et al.* Generation of surface plasmons at single subwavelength slits: from slit to ridge plasmon. New J. Phys. 10, 105014 (2008).

[b26] Sun W., He Q., Sun S. & Zhou L.. High-efficiency surface plasmon meta-couplers: concept and microwave-regime realizations. Light: Sci. Appl. 5, el6003 (2016). 10.1038/lsa.2016.3 PMC6059849 30167110

[b27] Shen X. & Cui T. J.. Planar plasmonic metamaterial on a thin film with nearly zero thickness. Appl. Phys. Lett. 102, 211909 (2013).

[b28] Gan Q., Fu Z., Ding Y. J. & Bartoli F. J.. Bidirectional subwavelength slit splitter for THz surface plasmons. Opt. Express 15, 18050–18055 (2007). 19551102 10.1364/oe.15.018050

[b29] López-Tejeira F. *et al.* Efficient unidirectional nanoslit couplers for surface plasmons. Nat. Phys. 3, 324–328 (2007).

[b30] Lin J. *et al.* Polarization-controlled tunable directional coupling of surface plasmon polaritons. Science 340, 331–334 (2013). 23599488 10.1126/science.1233746

[b31] Li X., Tan Q., Bai B. & Jin G.. Experimental demonstration of tunable directional excitation of surface plasmon polaritons with a subwavelength metallic double slit. Appl. Phys. Lett. 98, 251109 (2011).

[b32] Xu T. *et al.* Directional excitation of surface plasmons with subwavelength slits. Appl. Phys. Lett. 92, 101501 (2008).

[b33] Wang Y. *et al.* A tunable unidirectional surface plasmon polaritons source. Opt. Express 17, 20457–20464 (2009). 19997274 10.1364/OE.17.020457

[b34] Wang J. *et al.* Splitting and unidirectional excitation of surface plasmon polaritons by two uniform metallic nanoslits with a nanocavity antenna. J. Mod. Optic. 57, 1630–1634 (2010).

[b35] Kim H. & Lee B.. Unidirectional surface plasmon polariton excitation on single slit with oblique backside illumination. Plasmonics 4, 153–159 (2009).

[b36] Tang H.-H. & Liu P.-K.. Terahertz spoof surface plasmon polaritons unidirectional coupling via slit pair. Radio Science Conference (URSI AT-RASC), 2015 1st URSI Atlantic. IEEE, 1–1 (2015).

[b37] Kihm H. W., Lee K. G., Kim D. S., Kang J. H. & Park Q.-H.. Control of surface plasmon generation efficiency by slit-width tuning. Appl. Phys. Lett. 92, 051115 (2008).

[b38] Ahn K. J. *et al.* Optical and terahertz near-field studies of surface plasmons in subwavelength metallic slits. New J. Phys. 10, 105003 (2008).

[b39] Chen S., Jin S. & Gordon. R. Subdiffraction focusing enabled by a Fano resonance. Phys. Rev. X 4, 031021 (2014).

[b40] Lee K.-L., Wu S.-H., Lee C.-W. & Wei. P.-K. Sensitive biosensors using Fano resonance in single gold nanoslit with periodic grooves. Opt. Express 19, 24530–24539 (2011). 22109480 10.1364/OE.19.024530

[b41] Liu Z., Lee H., Xiong Y., Sun C. & Zhang. X. Far-field optical hyperlens magnifying sub-diffraction-limited objects, Science 35, 1686 (2007). 17379801 10.1126/science.1137368

[b42] Durant S., Liu Z., Steele J. M. & Zhang X.. Theory of the transmission properties of an optical far-field superlens for imaging beyond the diffraction limit. J. Opt. Soc. Am. B 23, 2383–2392 (2006).

[b43] Tuniz A. *et al.* Metamaterial fibres for subdiffraction imaging and focusing at terahertz frequencies over optically long distances. Nat. Commun. 4, 2706 (2013). 24162458 10.1038/ncomms3706 PMC3826642

[b44] Simovski C. R., Viitanen A. J. & Tretyakov S. A.. Sub-wavelength resolution in linear arrays of plasmonic particles. J. Appl. Phys. 101, 123102 (2007).

[b45] Cai1 B. G. *et al.* Leaky-Wave Radiations by Modulating Surface Impedance on Sub-wavelength Corrugated Metal Structures. Sci. Rep. 6, 23974 (2016). 27035269 10.1038/srep23974 PMC4817506

